# Accuracy and Precision of Iodine Quantification in Subtracted Micro-Computed Tomography: Effect of Reconstruction and Noise Removal Algorithms

**DOI:** 10.1007/s11307-023-01810-z

**Published:** 2023-04-03

**Authors:** Lízbeth Ayala-Dominguez, Luis-Alberto Medina, Carmen Aceves, Marcela Lizano, Maria-Ester Brandan

**Affiliations:** 1 Departamento de Física Experimental, Instituto de Física, Universidad Nacional Autónoma de México, Circuito de La Investigación Científica, Ciudad Universitaria UNAM, Mexico City, 04510 Mexico; 2 Department of Medical Physics, University of Wisconsin, 1111 Highland Ave, WI Madison, 53705 USA; 3 Unidad de Investigación Biomédica en Cáncer INCan-UNAM, Instituto Nacional de Cancerología, Av. San Fernando 22, Tlalpan, Mexico City, 14080 Mexico; 4 Departamento de Neurobiología Celular Y Molecular, Instituto de Neurobiología, Universidad Nacional Autónoma de México, Boulevard Juriquilla 3001, Querétaro Juriquilla, 76230 Mexico; 5 Departamento de Medicina Genómica Y Toxicología Ambiental, Instituto de Investigaciones Biomédicas, Universidad Nacional Autónoma de México, Circuito Exterior S/N, Ciudad Universitaria UNAM, Mexico City, 04510 Mexico

**Keywords:** Accuracy, Precision, Iodine concentration, Quantitative imaging, Micro-CT, Bilateral filter, Iterative reconstruction, SIRT

## Abstract

**Purpose:**

To evaluate the effect of reconstruction and noise removal algorithms on the accuracy and precision of iodine concentration (C_I_) quantified with subtracted micro-computed tomography (micro-CT).

**Procedures:**

Two reconstruction algorithms were evaluated: a filtered backprojection (FBP) algorithm and a simultaneous iterative reconstruction technique (SIRT) algorithm. A 3D bilateral filter (BF) was used for noise removal. A phantom study evaluated and compared the image quality, and the accuracy and precision of C_I_ in four scenarios: filtered FBP, filtered SIRT, non-filtered FBP, and non-filtered SIRT. *In vivo* experiments were performed in an animal model of chemically-induced mammary cancer.

**Results:**

Linear relationships between the measured and nominal C_I_ values were found for all the scenarios in the phantom study (R^2^ > 0.95). SIRT significantly improved the accuracy and precision of C_I_ compared to FBP, as given by their lower bias (adj. p-value = 0.0308) and repeatability coefficient (adj. p-value < 0.0001). Noise removal enabled a significant decrease in bias in filtered SIRT images only; non-significant differences were found for the repeatability coefficient. The phantom and *in vivo* studies showed that C_I_ is a reproducible imaging parameter for all the scenarios (Pearson r > 0.99, p-value < 0.001). The contrast-to-noise ratio showed non-significant differences among the evaluated scenarios in the phantom study, while a significant improvement was found in the *in vivo* study when SIRT and BF algorithms were used.

**Conclusions:**

SIRT and BF algorithms improved the accuracy and precision of C_I_ compared to FBP and non-filtered images, which encourages their use in subtracted micro-CT imaging.

**Supplementary information:**

The online version contains supplementary material available at 10.1007/s11307-023-01810-z.

## Introduction

Iodine concentration (C_I_) is a commonly assessed feature in quantitative contrast-enhanced computed tomography (CECT). It has been suggested as an imaging biomarker for evaluating treatment response and tissue vascularity and distinguishing among histological tumor subtypes [1–3]. Recent studies have evaluated its accuracy and precision under several imaging conditions [4–7]. Some studies have shown that bias, a measure of accuracy, increases with phantom size and decreases with radiation dose. Other studies have shown that the repeatability coefficient, a measure of precision, decreases with radiation dose and when iterative reconstruction (IR) algorithms are used, compared to the commonly used filtered backprojection (FBP) algorithm. These rigorous evaluations are not only useful but necessary to fully characterize the properties of C_I_ as a quantitative imaging biomarker.

Accurate and precise C_I_ values are essential to enable reliable and reproducible detection and quantification tasks in the clinical and preclinical scenarios. This is the case when small changes or thresholds in C_I_ are used to assess changes during treatment or post-treatment [1, 4], to distinguish between pathological types or risk subgroups [3, 7], for staging or evaluating tumor burden and metastasis [7], to evaluate tumor heterogeneity or perfusion parameters from C_I_ maps or time-C_I_ curves [1–3]. Moreover, the evaluation of accuracy and precision are key features in the current guidelines for the translation of quantitative imaging biomarkers into the clinic, as well as for the optimization and standardization of image acquisition and analysis, or to understand their limitations [8–10].

C_I_ has been evaluated in the preclinical setting, particularly for the study of animal models of cancer with contrast-enhanced micro-computed tomography (CE micro-CT) to estimate the angiogenic status of tumors or their vascularization [11–17]. Recently, one study has reported the evaluation of the bias of C_I_ to compare the performance of two imaging detectors under two CE micro-CT imaging protocols [16]. To the best of our knowledge, no other studies have evaluated the accuracy or precision of C_I_ in CE micro-CT. Additionally, current approaches in CE micro-CT involve the use of iterative reconstruction and noise removal algorithms that could have an impact on the accuracy and precision of C_I_ [15, 18–20]. The findings in CECT and the current approaches in CE micro-CT highlight the necessity to evaluate the impact of both conventional and novel approaches in CE micro-CT on the accuracy and precision of C_I_.

The aim of this work was to assess the accuracy and precision of C_I_ quantified in CE micro-CT images reconstructed and post-processed under several conditions: images reconstructed with the micro-CT vendor’s conventional reconstruction algorithm (FBP-based), images reconstructed with an in-house-implemented IR algorithm, and images filtered with a noise removal algorithm. The noise removal algorithm was a 3D bilateral filter (BF) specifically designed for either the FBP or IR algorithm. A phantom study evaluated and compared image quality, and the accuracy and precision of C_I_ quantified under these conditions. An animal study assessed the *in vivo* performance of C_I_ under the same conditions. All images were acquired with a previously optimized protocol for the assessment of vascular parameters with subtracted CE micro-CT imaging [21], and the IR and BF algorithms had also been previously validated and reported [20, 22].

## Materials and Methods

### Image Acquisition and Reconstruction

Image acquisition was performed with the micro-CT scanner of the trimodal PET/SPECT/CT Albira ARS preclinical system (Bruker, Spain). Micro-CT images were acquired with 45 kV, 0.8 mA, and 400 projections, according to a previously optimized imaging protocol for subtracted CE micro-CT imaging with this scanner [15]. This protocol involved the acquisition of two images: a pre-contrast image (i.e., the baseline image) and a second image after or during the administration of the contrast agent (i.e., the contrast-enhanced (CE) image). The baseline image was then subtracted from the CE image to yield the subtracted CE image, which was then parameterized to units of C_I_ with a calibration function to yield the subtracted C_I_ image. The total radiation dose to water measured at the isocenter for this imaging protocol was 680 mGy [15].

**Fig. 1:**
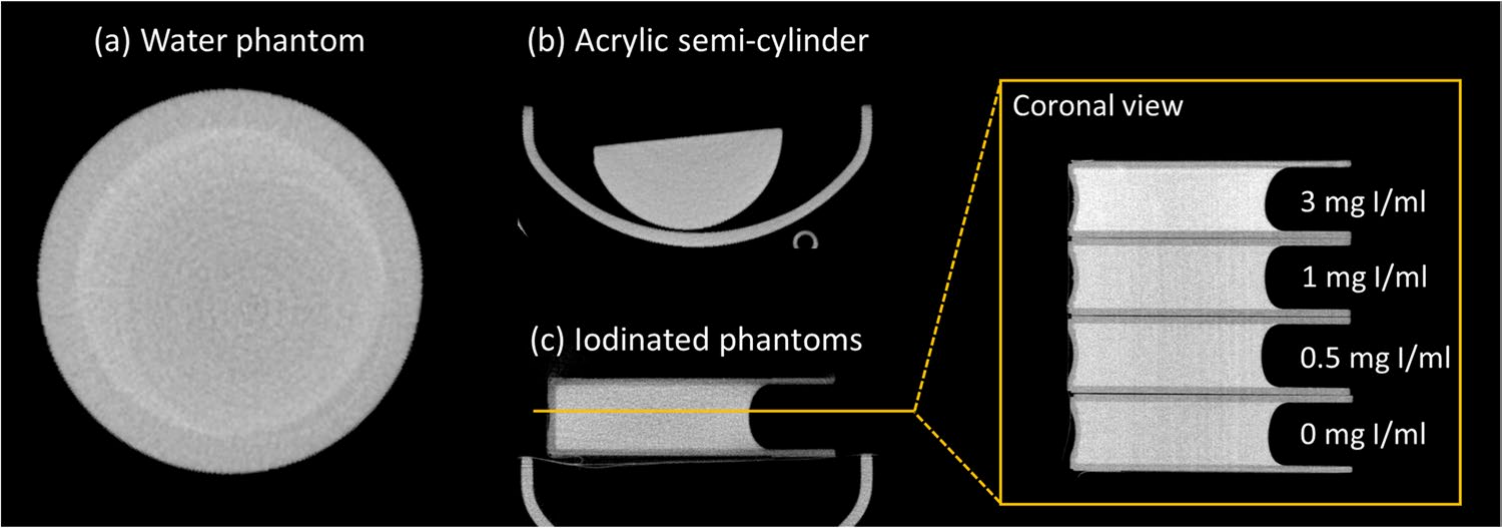
(**a**) Water phantom, (**b**) acrylic semi-cylinder phantom, and (**c**) calibrated iodinated phantoms

Image reconstruction was performed with either the conventional vendor’s reconstruction algorithm (FBP-based) or with a simultaneous iterative reconstruction technique (SIRT) algorithm implemented in-house with the Matlab R2018b (The MathWorks Inc., Natick, MA, USA) ASTRA toolbox. The SIRT algorithm was previously validated and optimized [22]. The number of iterations for the SIRT algorithm was optimized in this study as a trade-off between noise and spatial resolution, as described in the Supplementary Appendix and briefly summarized here. Increasing the number of iterations increased the noise and improved the spatial resolution in SIRT reconstructed images, as shown in Supplementary Figure S1. A range of 85–180 iterations were evaluated, and 180 iterations were selected since this number produced SIRT images with the highest spatial resolution among the evaluated number of iterations. An adverse consequence of selecting a high number of iterations, however, is that a higher noise content would be observed in the SIRT images used in this study, compared to the FBP images. FBP reconstructed images had a matrix size of 560 × 560x516 and a pixel size of 0.125 mm; SIRT reconstructed images had a matrix size of 750 × 750x657 and a pixel size of 0.1 mm. Images were calibrated to Hounsfield units (HU) using the average attenuation value of water for each reconstruction algorithm, which was obtained from images of a water phantom; a transverse view of this phantom is shown in Fig. 1.

### Noise Removal

A BF is a spatial-domain non-linear function designed to reduce noise while preserving the small structures and edges [23]. Its optimal parameters are related to the noise and spatial resolution of the image to be filtered, which means that they are unique for a given imaging protocol and reconstruction algorithm. In this work, a specific 3D BF was used for micro-CT images reconstructed with SIRT or FBP algorithms. BFs were implemented in MATLAB R2018b (The MathWorks Inc., Natick, MA, USA); the details of their implementation have been described previously [20].

### Quantification of Image Quality

Image quality was assessed with the noise power spectrum (NPS), the modulation transfer function (MTF), and the contrast-to-noise ratio (CNR) in four scenarios: FBP reconstructed images, SIRT reconstructed images, filtered FBP images (fFBP), and filtered SIRT images (fSIRT). All images were reconstructed with the FBP and SIRT algorithms. The fFBP and fSIRT images were obtained after applying the corresponding BF to the reconstructed images. The 2D and 1D NPS were evaluated from images of a water phantom, and the 1D MTF was evaluated in the transverse plane from images of an acrylic semi-cylinder phantom, following guidelines for the assessment of image quality in CT scanners [24, 25]. Figure 1 shows the water and acrylic phantoms.

Calibrated iodinated phantoms were used to assess the CNR[26]. These phantoms consisted of a solid epoxy material with C_I_ values of 0, 0.5, 1.0 and 3.0 mg I/ml, as shown in Fig. 1. One image of each iodinated phantom was acquired in a separate and consecutive manner with the same acquisition parameters, as defined above. An affine registration was performed between each image and the image of the 0 mg I/ml phantom [27], which was considered the baseline image, and then the baseline image was subtracted from the images of the other iodinated phantoms to yield the subtracted CE images.

The contrast was quantified from the subtracted CE images as the difference between each iodinated phantom and the 0 mg I/ml phantom. The noise was evaluated as the standard deviation of the mean value measured in the subtracted CE image of the 0 mg I/ml phantom. The CNR was estimated as the contrast of each iodinated phantom divided by the noise. The NPS, MTF, and CNR were quantified using MATLAB R2018b (The MathWorks Inc., Natick, MA, USA).

### Accuracy and Precision of C_I_: Phantom study

The subtracted CE images were converted to C_I_ using an appropriate calibration function for each reconstruction algorithm, to yield the C_I_ images. The calibration functions (C_I_ vs. HU measured in the subtracted CE images of the calibrated iodinated phantoms) were C_I_ = 0.022*CE + 0.298 (R^2^ = 0.99) and C_I_ = 0.023*CE – 0.095 (R^2^ = 0.99), for FBP and SIRT images, respectively.

Three repeated measurements of C_I_ were performed for each nominal C_I_ value. A separate set of images of the iodinated phantoms was acquired in a second experiment to assess the precision with a test–retest approach; C_I_ values were measured in this set of images to yield the replicate measurements.

The linear relationship between the measured and the nominal C_I_ values were assessed for the FBP, SIRT, fFBP, and fSIRT images [10]. Plots of the replicate measurements were obtained (measured vs. nominal C_I_); 2nd and 1st order polynomials were fitted to the data, and linearity was supported when the β_2_ coefficient of the 2nd order term of the 2nd order fitted polynomial was small (β_2_ < 0.5), and the β_1_ coefficient of the 1st order term of the 1st order fitted polynomial was close to one (0.95 < β_1_ < 1.05) and R^2^ > 0.9. The C_I_ accuracy was assessed with the bias [10], which was determined as the difference between the measured value and the nominal value; the bias was plotted against the nominal value.

The precision of C_I_ was estimated from repeatability and reproducibility metrics [10]. Repeatability was assessed with the within-subject standard deviation (wSD = standard deviation of the replicate measurements for each nominal C_I_ value), the within-subject coefficient of variation (wCV = wSD/mean), and the repeatability coefficient (RC = 2.77wSD). The reproducibility of C_I_ was evaluated with the correlation coefficient for the following comparisons: FBP vs. SIRT, FBP vs. fFBP, SIRT vs. fSIRT, and fFBP vs. fSIRT.

### *In vivo* Evaluation

All experimental procedures with the animals were reviewed and approved by the Ethics Committee and the Institutional Animal Care and Use Committee of the Instituto Nacional de Cancerología, Mexico, where all the experiments took place; approval number: (018/051/IBI) (CEI/1294/18). The *in vivo* evaluation was performed on a virgin female Sprague–Dawley rat with chemically-induced mammary cancer. Mammary lesions were chemically induced with dimethylbenz[a]anthracene (DMBA) [28]. The animal was kept in a pathogen-free environment and fed with autoclaved food and water *ad libitum*. A single intragastric dose of 20 mg/ml DMBA (Sigma) dissolved in 1 ml of sunflower oil was administered to the animal (7-week-old), after a previous intraperitoneal injection of ketamine and xylazine (30 and 6 mg/kg body weight, respectively) [28]. Imaging was performed after tumor detection, which occurred 10 weeks after the inoculation of DMBA. For image acquisition, the animal was anesthetized with isoflurane (3% in 100% oxygen). A baseline image was acquired; then, a CE image was acquired during continuous infusion of a clinical contrast agent (Omnipaque 300, GE Healthcare,Wauwatosa, WI, USA; average dose = 2.4 mg of iodine/g of body weight (b.w.), infusion rate = 0.5 mL/min), via a catheter placed in the right external jugular vein of the animal. No gating (cardiac or respiratory) was used during image acquisition. Images were reconstructed, filtered, registered, and subtracted as described and converted to C_I_ values. Mean C_I_ and its standard deviation were quantified in FBP, SIRT, fFBP, and fSIRT images within spherical volumes of interest (VOIs) with AMIDE software [29] for several tissues. VOIs were placed in the left ventricle (LV, 3 mm diameter), abdominal aorta (0.7 mm diameter), liver (3 mm diameter), tumor (2 mm diameter), and muscle (2 mm diameter). A CNR related to muscle (CNR_muscle_) was obtained for each tissue; in this case, the contrast was evaluated as the difference between C_I_ within each tissue and C_I_ within the muscle, and the noise was defined as the standard deviation of the mean value of C_I_ within the muscle.

### Statistical Analysis

GraphPad Prism 6 (GraphPad Software, Inc., San Diego, CA, USA) was used to perform all statistical analyzes. A Shapiro–Wilk test was used to assess the normality of the data. Data were compared in the following pairs: FBP vs. SIRT, FBP vs. fFBP, SIRT vs. fSIRT, and fFBP vs. fSIRT. Normally distributed data were compared with a one-way analysis of variance (ANOVA) test, followed by Bonferroni’s test for multiple comparisons (namely, CNR, bias, RC, CNR_muscle_). Non-parametric data were compared with the Friedman test and Dunn’s multiple comparison test (namely, NPS, MTF, C_I_ in the *in vivo* evaluation). Pearson correlation coefficient was used to evaluate the reproducibility of C_I_. An adjusted p-value less than 0.05 was considered as statistically significant.

## Results

### Image Quality

**Fig. 2:**
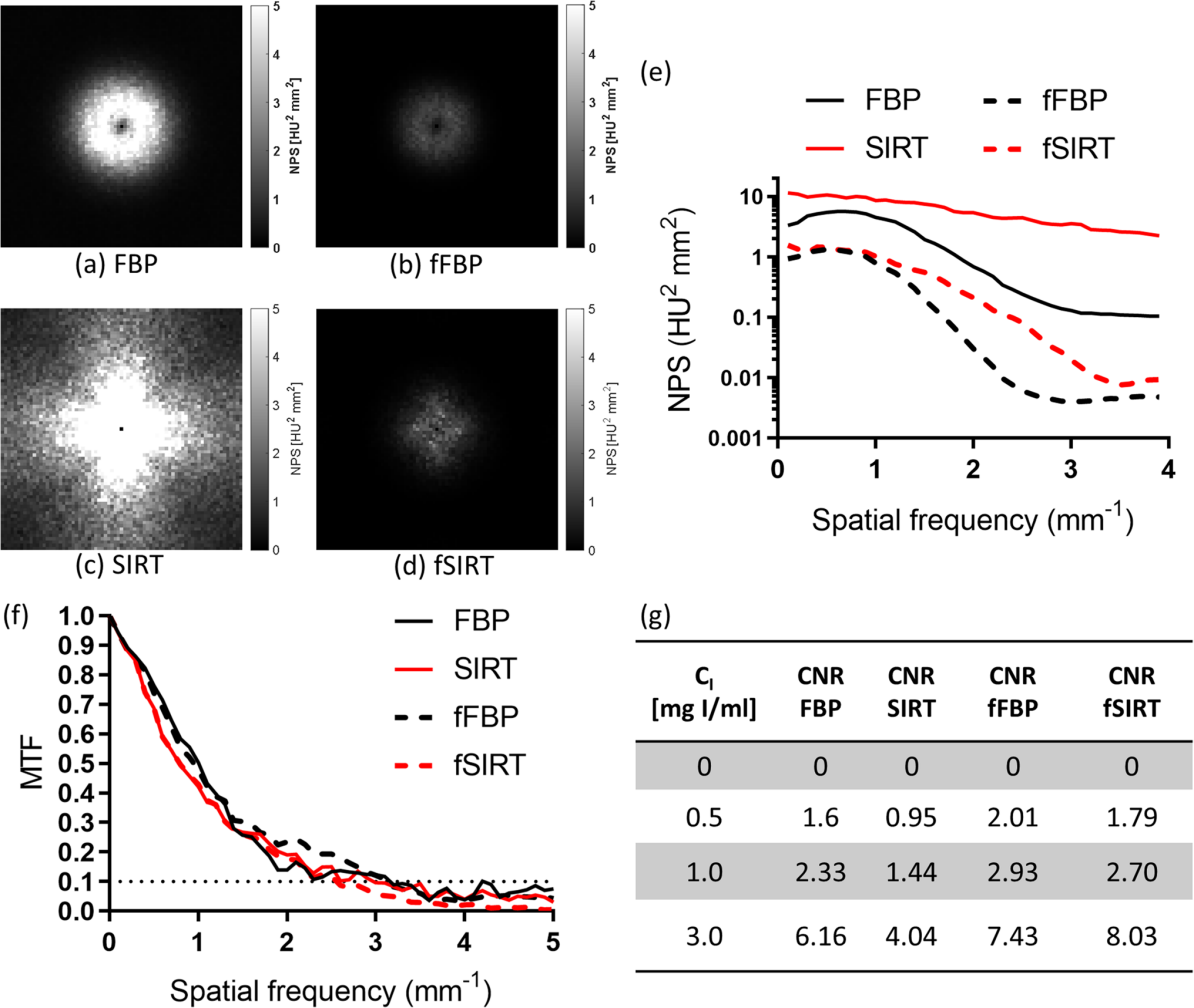
2D Noise power spectrum (NPS) for (**a**) FBP images: images reconstructed with a commonly used algorithm (filtered backprojection); (**b**) fFBP images: FBP images after the application of the bilateral filter (BF); (**c**) SIRT images: images reconstructed with an iterative algorithm (simultaneous iterative reconstruction technique), and (**d**) fSIRT images: SIRT images after the application of the BF. Image quality was assessed by the (**e**) 1D NPS, (**f**) the modulation transfer function (MTF), and (**g**) the contrast-to-noise ratio (CNR) for several iodine concentrations (**C**_I_)

Figure 2 shows the results of the evaluation of image quality for FBP, SIRT, fFBP, and fSIRT images. The 2D NPS in Fig. 2a-d show isotropically distributed values for FBP and fFBP images, and anisotropically distributed values for SIRT and fSIRT images. This difference reflects the non-linearity of the SIRT algorithm, compared to the linear FBP algorithm. As can be observed in Fig. 2a-d, the BF reduces the amplitude of the noise for both reconstruction algorithms. As shown in Fig. 2e, the noise removal was more marked for the SIRT algorithm compared to FBP, and both algorithms showed a change in texture, since the peak and form of the 1D NPS curve were lost after the filtration. Statistically significant differences were found between the NPS mean values for FBP vs. SIRT (adjusted -value = 0.0025), FBP vs. fFBP (adj. p-value < 0.0001), SIRT vs. fSIRT (adj. p-value < 0.0001), and fFBP vs. fSIRT (adj. p-value = 0.0047), evaluated with the Friedman test. It is known that iterative algorithms, particularly SIRT, yield images with lower noise than images obtained with FBP [30]. However, the higher noise in SIRT images compared to FBP images observed in Fig. 2e is related to the number of iterations chosen in this work for the SIRT algorithm.

Figure 2f shows the MTF for the scenarios evaluated. Although the values were similar, a statistically significant difference was found for FBP vs. SIRT (adj. p-value = 0.0051), and fFBP vs. fSIRT (adj. *p*-value < 0.0001), evaluated with the Friedman test. The agreement found between FBP vs. fFBP and SIRT vs. fSIRT, and the 1D NPS results, demonstrates the adequate functioning of the BF: it reduces image noise while it preserves the spatial resolution.

Figure 2g shows the CNR at the evaluated nominal C_I_ values for FBP, SIRT, fFBP, and fSIRT images. Non-significant statistical differences were found when comparing these results, despite the fact that CNR was higher in the filtered images compared to the non-filtered images.

### Accuracy and Precision of C_I_

**Fig. 3:**
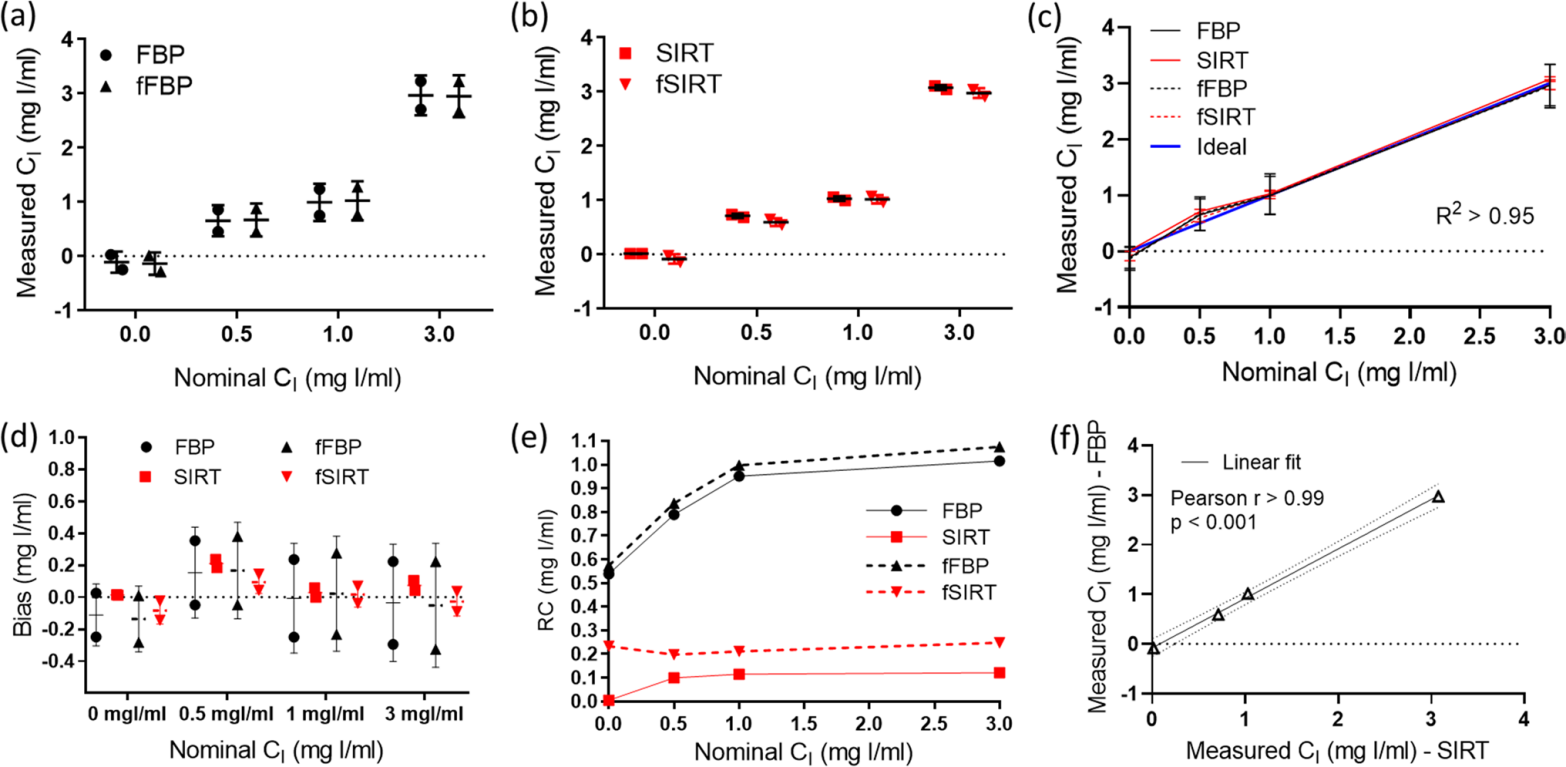
Comparison of measured and nominal iodine concentration (C_I_) values from (**a**) FBP and (**b**) SIRT images. (**c**) Evaluation of the relationship between measured and nominal C_I_ values. Assessment of the accuracy and precision of C_I_ with (**d**) bias, (**e**) the repeatability coefficient (RC), and (**f**) the reproducibility of C_I_ quantified in FBP vs. SIRT images (evaluated with the Pearson correlation coefficient)

Figure 3a and b show the replicate C_I_ measurements compared to the nominal C_I_ values for FBP and SIRT images; a higher variability was observed in the replicate C_I_ measurements for the FBP images compared to the SIRT images. A linear relationship (R^2^ > 0.95) was found between the measured and nominal C_I_ values for the evaluated scenarios, as shown in Fig. 3c, which reflects the similarity between the measured and nominal C_I_ values.

Figure 3d shows the bias for FBP, SIRT, fFBP, and fSIRT images. Statistically significant differences were found for FBP vs. SIRT (adj. p-value = 0.0308) and SIRT vs. fSIRT (adj. p-value = 0.0308), which suggests that the use of the SIRT algorithm compared to the FBP algorithm has an impact on the accuracy of C_I_, while the use of the BF only affects the accuracy in SIRT images.

Figure 3e shows the RC for FBP, SIRT, fFBP, and fSIRT images. A statistically significant difference was found for FBP vs. SIRT (adj. p-value < 0.0001) and fFBP vs. fSIRT (adj. p-value < 0.0001), which suggests that the use of the SIRT algorithm compared to the FBP algorithm increases the precision of C_I_, however, its precision is not affected by using the BF in either the FBP or SIRT images.

Figure 3f shows an example of the evaluation of the correlation between measured C_I_ values in FBP and SIRT images; strong associations were found for all the comparisons (Pearson r > 0.99, p-value < 0.001). These results suggest that C_I_ values are reproducible across the reconstruction and noise removal algorithms in the evaluated C_I_ range.

### *In vivo *Evaluation of C_I_

**Fig. 4:**
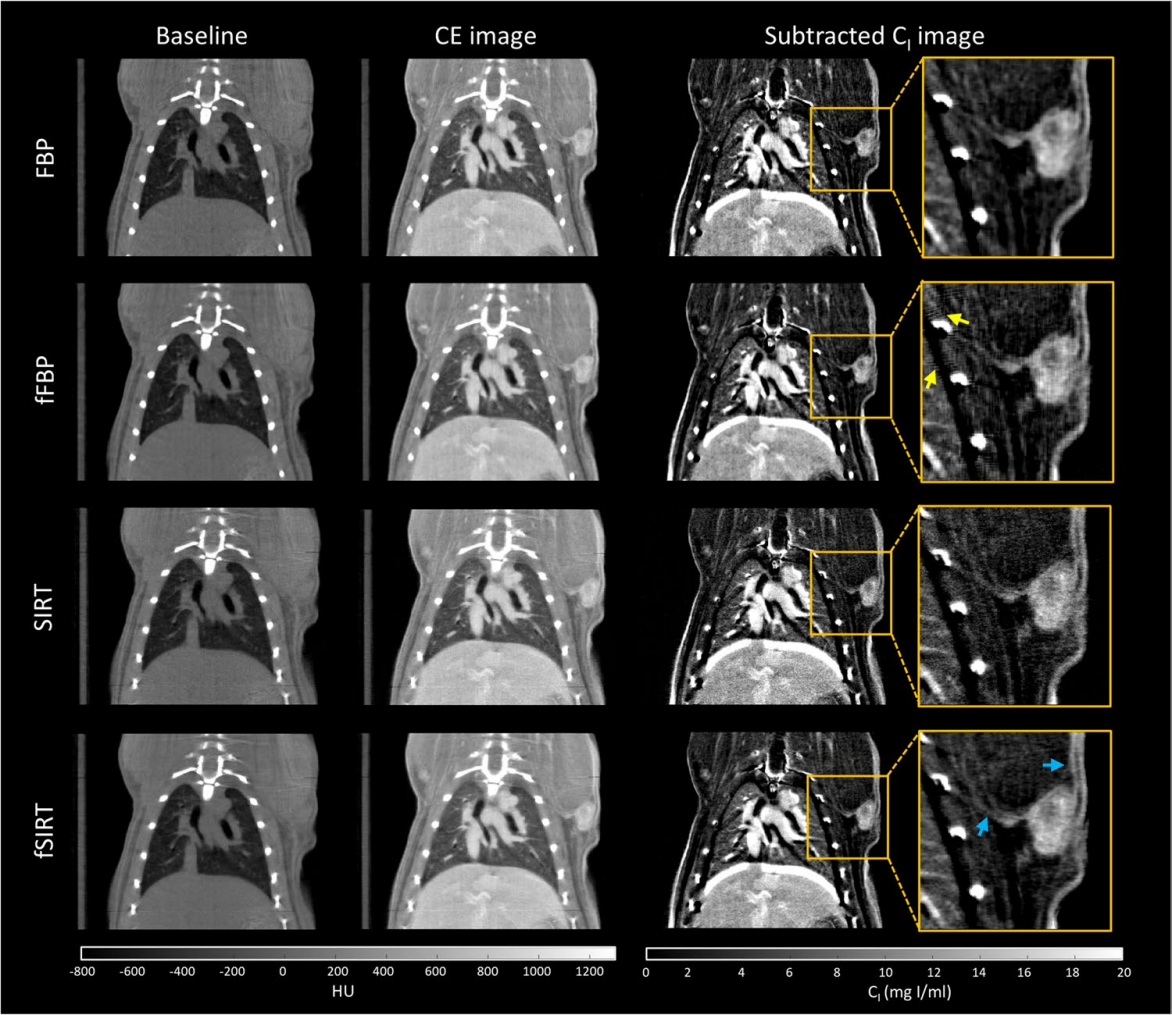
Coronal views of baseline, contrast-enhanced (CE), and subtracted micro-CT images of an animal model of chemically-induced mammary cancer. No gating was used during image acquisition. Images were reconstructed with an FBP or SIRT algorithm and filtered with a bilateral filter (fFBP and fSIRT, respectively). Subtracted images are shown in units of iodine concentration (C_I_). The yellow arrows show artifacts in the fFBP images; the blue arrows show tumor vessels. HU: Hounsfield units

Figure 4 shows the coronal views of baseline, CE, and subtracted C_I_ images of an animal model of chemically-induced mammary cancer; a magnification of the tumor region is shown in the insets. As shown in Fig. 4, subtracted C_I_ images enabled a better depiction of the tumor regions with high and low C_I_ values and their vasculature, compared to baseline and CE images for all the evaluated scenarios. Qualitatively, SIRT images showed a higher noise content than FBP images, as well as a slightly better spatial resolution indicated by the better definition of some structures like the ribs of the animal; these results agree with the quantitative evaluation of image quality shown in Fig. 2. The yellow arrows in Fig. 4 indicate the enhancement of the streak artifacts present in the FBP images as a result of the use of the BF. The enhancement of these artifacts is observed as thicker alternating black lines. The blue arrows in Fig. 4 indicate a slightly better definition of the vasculature in SIRT images after the application of the BF (fSIRT image), compared to the SIRT images, despite no significant difference being found in the MTF for these images. A bright artifact can be observed in the boundary between the lungs and the liver of the animal in the subtracted C_I_ image in Fig. 4, due to the misregistration caused by respiratory motion (no gating was used during image acquisition). If respiratory gating were used, it could improve the quality of the subtraction and potentially reduce this artifact [17].

**Fig. 5:**
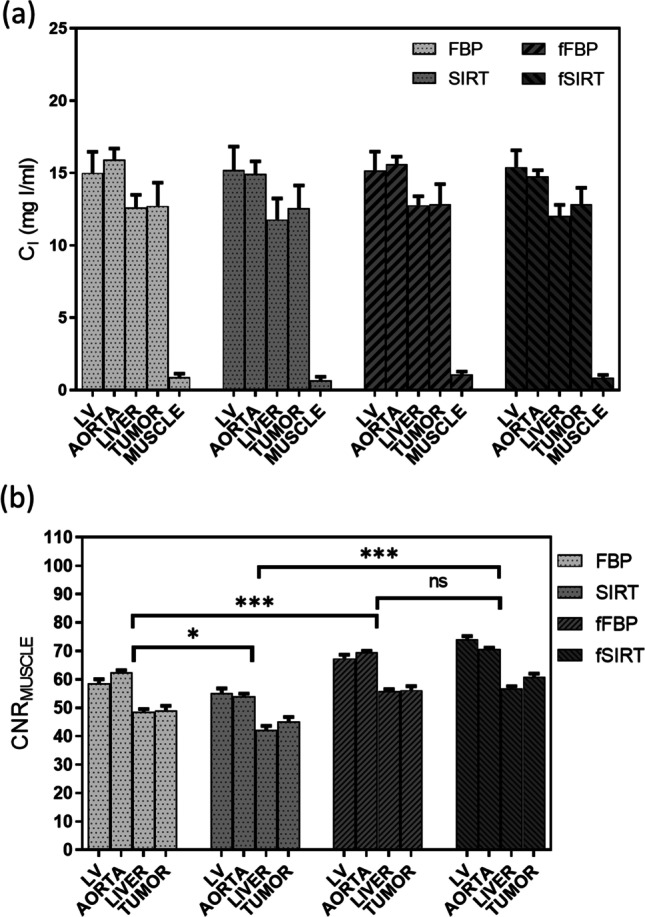
*In vivo* quantitative assessment of (**a**) iodine concentration (C_I_) and (**b**) image quality, assessed with the contrast-to-noise ratio related to muscle (CNR_muscle_). LV = left ventricle, ns = non-significant, *Adjusted p-value = 0.0168, ***adj. p-valued = 0.0003; evaluated with one-way ANOVA and Bonferroni’s test for multiple comparison

Figure 5a shows the quantitative *in vivo* evaluation of C_I_ in several tissues in FBP, SIRT, fFBP, and fSIRT images. An agreement was found among the measured C_I_ values for each scenario, which agrees with the reproducibility results exemplified in Fig. 3f. Figure 5b shows the CNR_muscle_, which was quantified from the contrast observed between each tissue and muscle. Statistically significant differences were found in CNR_muscle_ quantified in FBP vs. SIRT (adj. p-value = 0.0168), FBP vs. fFBP (adj. p-value = 0.0003), and SIRT vs. fSIRT (adj. p-value = 0.0003), evaluated with one-way ANOVA, which suggest that filtered images, regardless of the reconstruction algorithm, showed a higher image quality. This result highlights the importance of performing the *in vivo* evaluations since the phantom study showed that the difference in CNR was not statistically significant.

## Discussion

In this work, we have evaluated the effect of reconstruction (by FBP-based vendor’s and in-house SIRT algorithms) and noise removal (by a 3D BF algorithm) on the accuracy and precision of C_I_ quantified with subtracted CE micro-CT. A phantom study evaluated and compared image quality, and the accuracy and precision of C_I_ under four scenarios: FBP, SIRT, fFBP, and fSIRT images. *In vivo* experiments evaluated image quality and the reproducibility of C_I_ under the same scenarios in an animal model of chemically-induced mammary cancer.

The image quality evaluation in the phantom study showed a significant effect of the SIRT and BF algorithms on the image noise; however, this effect did not introduce significant changes in the CNR. Specifically, the BF reduced the noise and maintained the spatial resolution for the two reconstruction algorithms evaluated, as expected. As expected from the optimization of the number of iterations for the SIRT algorithm, the noise in SIRT images was higher than the noise in FBP images. The CNR was higher in the filtered images compared to the non-filtered images, however, this difference was non-significant. One of the main findings of this work was that the use of the SIRT algorithm significantly improved the accuracy and precision of C_I_ by reducing the bias and the RC, respectively, compared to the FBP algorithm. Interestingly, it was also found that the use of the BF maintained the improvement in the precision of C_I_ for the SIRT images, which encourages its use in subtracted CE micro-CT imaging. For the reproducibility, a high association was found among the scenarios evaluated, as demonstrated by the strong correlation coefficients found in this work for the evaluated C_I_ range.

The *in vivo* study demonstrated the reproducibility of C_I_ values quantified in images of the animal model. The main finding of the *in vivo* study was that a significant improvement in image quality was observed with the use of the BF, although non-significant differences were found in the phantom study. This significant result demonstrates the relevance of performing preliminary *in vivo* studies in the validation stage, since they involve biological variables that phantoms cannot usually resemble.

A direct comparison with previously reported findings is not possible since, to our knowledge, this is the first study that specifically addresses the effects of iterative reconstruction and noise removal algorithms on the accuracy and precision of C_I_ in subtracted CE micro-CT. However, some similarities can be found with studies performed in CECT imaging. In a phantom study, Euler et al. found that the accuracy of C_I_ depended on the scanner type, patient-related factors (such as size), radiation dose, and the reconstruction algorithm [4]. For the reconstruction algorithm, it was observed that the use of iterative algorithms (although different from SIRT) increased the accuracy of C_I_ compared to FBP, in agreement with our results for SIRT. In another phantom study, Chen et al. found that the precision of C_I_ was related to the radiation dose and the reconstruction algorithm [6]; it was observed that the RC decreased when IR algorithms were used compared to FBP, which agrees with our results.

Some authors have explored the effect of noise removal algorithms (different from IR algorithms) on image quality or the quantification of vascular parameters. Davidoiu et al. found that NPS decreased and CNR increased when different noise removal algorithms were applied to FBP-reconstructed micro-CT images of digital and physical phantoms [31]. Although the algorithms evaluated did not include the BF, in general, those findings agree with ours for fFBP images. In another study, Yeung et al. found similar image quality results both in a digital phantom and in an animal model of glioma when another noise removal and an IR algorithm were used [32]. Moreover, they found that the accuracy of perfusion parameters such as blood volume or blood flow increased when the noise removal algorithm was applied to images of a digital phantom, which is in general agreement with our results for C_I_ in fSIRT images. In the clinical scenario, Pisana et al*.* found a high similarity between ground truth values of blood volume and values obtained from FBP images filtered with a modified BF [33]. Additionally, Pisana et al. found a higher CNR in the filtered images compared to non-filtered images, which is in agreement with our results.

The present work has some limitations. First, the accuracy and precision of C_I_ were evaluated with calibrated phantoms with a limited concentration range (0–3 mg I/ml). This C_I_ range could be appropriate for tumor studies, since previous works have reported values of C_I_ < 5 mg I/ml in several animal cancer models [1, 11, 14, 15]. However, a wider range of C_I_ should be evaluated to extend the understanding of the accuracy and precision of C_I_ in several organs and tissues. Another limitation is that only one size of the calibrated phantom was evaluated, and it has been demonstrated that the accuracy of C_I_ depends on the phantom size [4]. Besides evaluating different-sized phantoms, a non-uniform background and contrast extravasation could be incorporated to simulate the complex structure of the *in vivo* studies [31]. The discrepancy found between the phantom and the *in vivo* results for the CNR could be related to this lack of complexity in the phantoms used in this study. Finally, although it has been demonstrated that acquisition parameters such as kilovoltage and radiation dose significantly impact the accuracy and precision of C_I_, those parameters were not considered in the present study since the imaging protocol had been previously optimized and standardized [21, 22]. This optimization was carried out in a systematic and rigorous manner, and it included the optimization of image quality and radiation dose.

## Conclusion

The improvements in accuracy and precision of C_I_ enabled by the SIRT and BF algorithms compared to FBP and non-filtered images encourages their use in subtracted micro-CT imaging.

## Conflict of Interest

Lizbeth Ayala-Domínguez, Luis Alberto Medina, and María Ester Brandan report a grant from Consejo Nacional de Ciencia y Tecnología (CONACyT), grant CB-251497, during the conduct of the study. Marcela Lizano and Carmen Aceves declare that there is no conflict of interest.

### Supplementary information

Below is the link to the electronic supplementary material.

Supplementary file1 (DOCX 724 KB)

## Data Availability

The data that support the findings of this study are available from the corresponding author upon reasonable request.
