# Supplementary material for: Accuracy and Precision of Iodine Quantification in Subtracted Micro-Computed Tomography: Effect of Reconstruction and Noise Removal Algorithms
Source: Mol Imaging Biol. 2023 Apr 3;25(6):1084–93. doi: 10.1007/s11307-023-01810-z (PMC10728260; doi:10.1007/s11307-023-01810-z)
Supplement: Supplementary file 1 — Supplementary file1 (DOCX 724 KB) [file 11307_2023_1810_MOESM1_ESM.docx]

**Electronic Supplementary Material**

**Accuracy and precision of iodine quantification in subtracted micro-computed tomography:**

**effect of reconstruction and noise removal algorithms**

**Journal: Molecular Imaging and Biology**

Lizbeth Ayala-Domínguez^1,2*^, Luis-Alberto Medina^1,3^, Carmen Aceves^4^, Marcela Lizano^3,5^, María-Ester Brandan^1^

^1^Departamento de Física Experimental, Instituto de Física, Universidad Nacional Autónoma de México, Circuito de la Investigación Científica, Ciudad Universitaria UNAM, Mexico City 04510, Mexico.

^2^Department of Medical Physics, University of Wisconsin, 1111 Highland Ave., Madison, Wisconsin 53705, United States.

^3^Unidad de Investigación Biomédica en Cáncer INCan-UNAM, Instituto Nacional de Cancerología, Av. San Fernando 22, Tlalpan, Mexico City 14080, Mexico.

^4^Departamento de Neurobiología Celular y Molecular, Instituto de Neurobiología, Universidad Nacional Autónoma de México, Boulevard Juriquilla 3001, Juriquilla, Querétaro 76230, Mexico.

^5^Departamento de Medicina Genómica y Toxicología Ambiental, Instituto de Investigaciones Biomédicas, Universidad Nacional Autónoma de México, Circuito Exterior S/N, Ciudad Universitaria UNAM, Mexico City 04510, Mexico.

*Corresponding author email: ayalaliz@gmail.com

**Supplementary Appendix**

**Optimization of the number of iterations for the SIRT algorithm**

The number of iterations used in the simultaneous iterative reconstruction technique (SIRT) influences image quality^1,2^. In our study, the number of iterations was optimized as a trade-off between noise and spatial resolution. Noise was quantified with the noise power spectrum (NPS) obtained from images of a water phantom, and spatial resolution was quantified with the modulation transfer function (MTF) obtained from images of an acrylic semi-cylinder, according to guidelines for the assessment of image quality in computed tomography^3,4^. Images were acquired with a trimodal PET/SPECT/CT Albira ARS preclinical system (Bruker, Spain), using 45 kV, 0.8 mA, and 400 projections. Images were reconstructed with the SIRT algorithm implemented in Matlab R2018b (The MathWorks Inc., Natick, MA, USA) with the ASTRA toolbox, and varying the number of iterations from 85 to 180. Images were also reconstructed with the vendor’s reconstruction algorithm, which is based on filtered back-projection (FBP).

Results showed that increasing the number of iterations increased the noise content in SIRT images, as shown in Figure S1a. Spatial resolution improved when the number of iterations increased, as shown in Figure S1b. Figure S1c shows these results in a qualitative manner for images of the water phantom and the acrylic semi-cylinder used to estimate the NPS and the MTF, respectively.


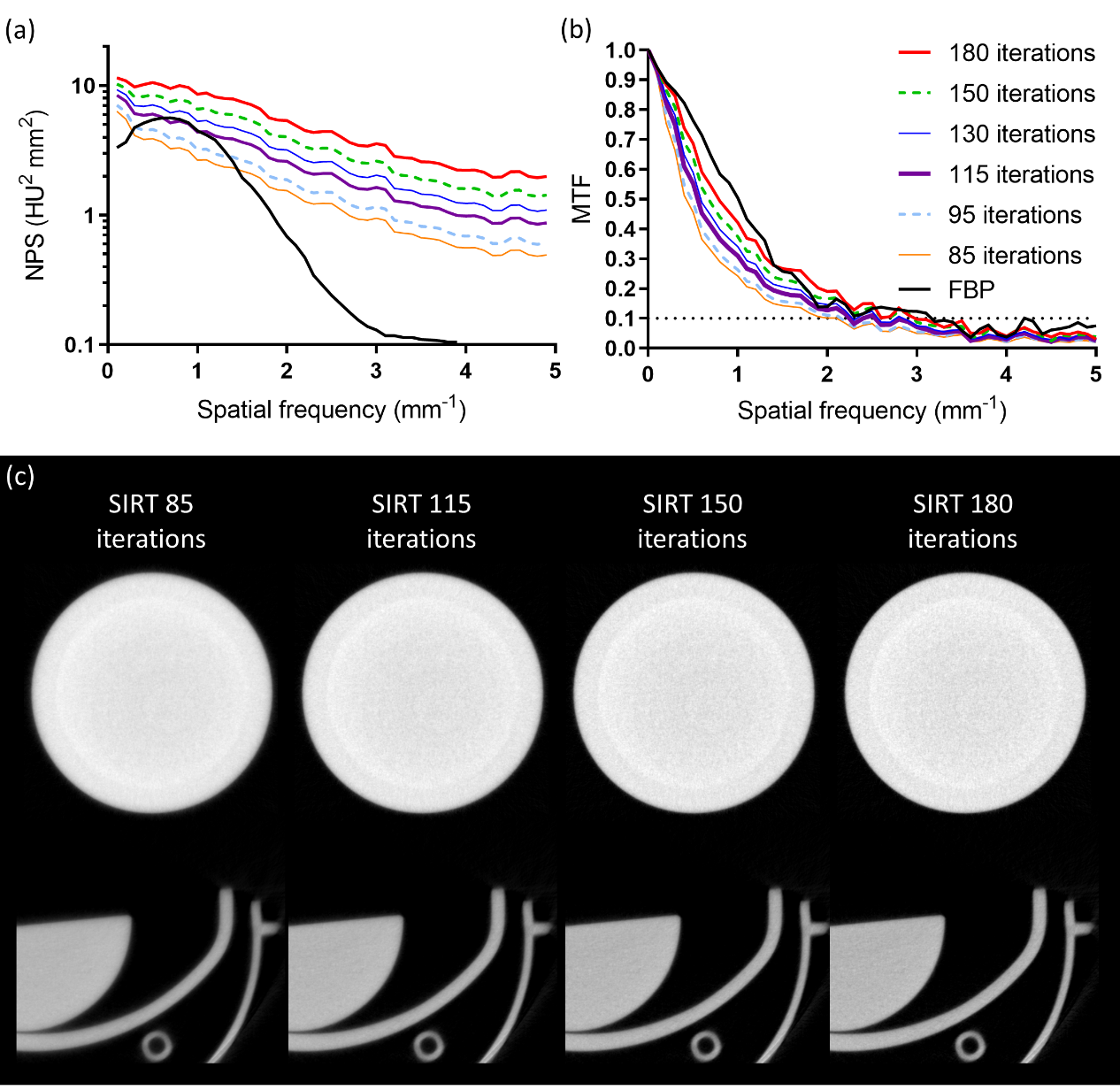


Figure S1. (a) NPS and (b) MTF of images reconstructed with the SIRT algorithm using different number of iterations; NPS and MTF are also shown for FBP images. (c) Qualitative demonstration of the effect of the number of iterations on noise and spatial resolution in images of the water and acrylic phantoms.

The lowest noise in SIRT images was found when 85 iterations were used, however, these images also showed the worst spatial resolution. The best spatial resolution was found when 180 iterations were used, however, these images also showed the highest noise content. Since spatial resolution is one of the most important parameters for the detection of blood vessels with *in vivo* preclinical imaging, 180 iterations were chosen to reconstruct SIRT images in our study.

**References:**

1. Pelt, D. M. & de Andrade, V. Improved tomographic reconstruction of large-scale real-world data by filter optimization. *Adv Struct Chem Imaging* **2**, (2017).

2. Kim, F. H., Pintar, A. L., Moylan, S. P. & Garboczi, E. J. The Influence of X-Ray Computed Tomography Acquisition Parameters on Image Quality and Probability of Detection of Additive Manufacturing Defects. *J Manuf Sci Eng* **141**, (2019).

3. Pahn, G., Skornitzke, S., Schlemmer, H. P., Kauczor, H. U. & Stiller, W. Toward standardized quantitative image quality (IQ) assessment in computed tomography (CT): A comprehensive framework for automated and comparative IQ analysis based on ICRU Report 87. *Physica Medica* **32**, 104–115 (2016).

4. Cassol, F. *et al.* Characterization of the imaging performance of a micro-CT system based on the photon counting XPAD3/Si hybrid pixel detectors. *Biomed Phys Eng Express* **2**, 025003 (2016).
